# Interventions for child development based on the Touchpoints Model: scoping review ^*^

**DOI:** 10.1590/1518-8345.6732.4035

**Published:** 2023-10-09

**Authors:** Jéssica Batistela Vicente, Talita Cristina Pegorin, Ana Laura de Oliveira Santos, Maria de La Ó Ramallo Veríssimo

**Affiliations:** 1 Universidade de São Paulo, Escola de Enfermagem, São Paulo, SP, Brasil.; 2 Becaria de la Coordenação de Aperfeiçoamento de Pessoal de Nível Superior (CAPES), Brasil.; 3 Becaria del Conselho Nacional de Desenvolvimento Científico e Tecnológico (CNPq), Brasil.

**Keywords:** Child Development, Parenting, Child, Family, Nursing, Review, Desarrollo Infantil, Responsabilidad Parental, Niño, Familia, Enfermería, Revisión, Desenvolvimento Infantil, Parentalidade, Criança, Família, Enfermagem, Revisão

## Abstract

**Objective:**

to map the characteristics of interventions to promote child development that used the Touchpoints Model.

**Method:**

this is a scoping review, guided by the recommendations of the JBI Reviewer’s Manual, carried out in nine databases, in the gray literature and in the reference list of the selected studies. The research question was “what are the characteristics of interventions with parents/caregivers and children to promote child development, from pregnancy to six years of age, based on the Touchpoints Model?”. Rayyan was used for the selection of studies and a standard form for data extraction. The analysis was carried out descriptively.

**Results:**

twelve publications were included in the review. Interventions were heterogeneous; concentrated in the period from pregnancy to three years of age; prioritized the dissemination of Touchpoints content according to age, and parenting aspects; most were performed by nurses, in Primary Health Care, and during home visits. Interventions were related to overall development, greater understanding of development and greater interaction with the child.

**Conclusion:**

studies have shown potential for favorable outcomes for child development and parenting. The variability of interventions made it difficult to map more effective characteristics.

## Abstract (es)

**Objetivo:**

mapear las características de las intervenciones para promover el desarrollo infantil que utilizaron el Modelo *Touchpoints*.

**Método:**

se trata de una revisión de alcance, guiada por las recomendaciones del JBI *Reviewer’s Manual*, realizada en nueve bases de datos, en la literatura gris y en la lista de referencias de los estudios seleccionados. La pregunta de investigación fue "¿cuáles son las características de las intervenciones con padres/cuidadores y niños para promover el desarrollo infantil, desde el embarazo hasta los seis años, con base en el Modelo *Touchpoints*?". Se utilizó el Rayyan para la selección de los estudios y un guión para la extracción de datos. El análisis se realizó de forma descriptiva.

**Resultados:**

se incluyeron doce publicaciones. Las intervenciones fueron heterogéneas; concentradas en el período comprendido entre el embarazo y los tres años de edad; priorizó la difusión del contenido de los *Touchpoints* según la edad y los aspectos de parentalidad; la mayoría fueron realizadas por enfermeros, en la Atención Primaria de Salud y durante las visitas domiciliarias. Las intervenciones se relacionaron con el desarrollo general, una mayor comprensión del desarrollo y una mayor interacción con el niño.

**Conclusión:**

los estudios han demostrado potencial para obtener resultados favorables para el desarrollo infantil y la parentalidad. La variabilidad de las intervenciones dificultó el mapeo de las características más efectivas.”

## Abstract (pt)

**Objetivo:**

mapear as características das intervenções para a promoção do desenvolvimento infantil que utilizaram o Modelo *Touchpoints*.

**Método:**

revisão de escopo, guiada pelas recomendações do JBI *Reviewer’s Manual* realizada em nove bases de dados, na literatura cinzenta e na lista de referências dos estudos selecionados. A questão de pesquisa foi: quais são as características das intervenções com pais/cuidadores e crianças para promoção do desenvolvimento infantil, da gestação até os seis anos de idade, baseadas no Modelo *Touchpoints*? Foram utilizados o Rayyan, para a seleção dos estudos, e um roteiro, para a extração de dados. A análise foi realizada de forma descritiva.

**Resultados:**

foram incluídas doze publicações. As intervenções eram heterogêneas; concentradas no período da gestação até os três anos de idade; priorizaram a disseminação de conteúdo dos *Touchpoints* segundo idade e aspectos de parentalidade; a maioria foi realizada por enfermeiros na Atenção Primária à Saúde e na visita domiciliar. As intervenções foram relacionadas ao desenvolvimento global, à maior compreensão sobre o desenvolvimento e à maior interação com a criança.

**Conclusão:**

os estudos evidenciaram potencial para resultados favoráveis ao desenvolvimento infantil e à parentalidade. A variabilidade das intervenções dificultou mapear características mais efetivas.

**Highlights:**

**(1)** Interventions in the period of pregnancy until the child’s 3 years of life predominated.

**(2)** Interventions by nurses in Primary Care and home visits predominated.

**(3)** The interventions were mostly delivered individually and face-to-face.

**(4)** The participating parents had a greater understanding of child development.

**(5)** There was more interaction with the child, use of toys and learning materials.

## Introduction

Early childhood is defined as the first six years of a child’s life, and has been recognized as a strategic moment to provide opportunities for the child’s biological, psychological, cognitive and social development ( 1 ). The implementation of interventions to promote development in this period of life has been a national and international priority. The Nurturing Care strategy, launched by the World Health Organization (WHO) in 2018 as a set of global actions aimed at responsive care for children, points to child development (CD) as an important aspect to achieve the Sustainable Development Goals (SDG- 2030) ( 2 ^-^ 3 ). 

Interventions aimed at CD have expanded knowledge about developmental science, and need to be implemented in a multisectoral way and anchored in nurturing care. The home environment and childcare environments represent a powerful and immediate nurturing context for children to reach their full developmental potential ( 1 ). 

In this context, evidence demonstrates that parenting interventions are necessary to promote healthy CD, being able to improve parental knowledge, parenting practices and interactions between parents and children ( 4 ^-^ 6 ). Parenting interventions also demonstrate positive effects on children’s cognitive, language, motor, socio-emotional and attachment development, results that are seen in low-, middle- and high-income countries ( 4 ). 

Programs focused on CD have improved child care, but there is a predominance of vertical interventions and family education, with scarce use of the theoretical framework to support them ( 7 ). 

A theoretical reference that has not yet been tested in the Brazilian reality and that aims at an intersectoral practice and with a focus on parental competence is the Touchpoints Model (TP). Created by Thomas Berry Brazelton, the TP Model is an approach to child development that is grounded in cultural sensitivity, reflective practice, and systems theory ( 8 ). This model establishes a way of caring for families by understanding the development and supporting family relationships. By adopting a new concept of child development - as non-linear, characterized by regressions, spurts and pauses - it understands that child behavior regressions can generate disorganization in the family system and be disruptive ( 9 ). The model addresses 15 Touchpoints, from prenatal care to 6 years old, which are periods of regression and disorganization of the child who is learning a new skill ( 9 ). 

In this approach, the family needs to be supported in a web of intersectoral relationships through an articulated work between health service professionals, educational and social sectors. The model seeks a paradigm shift to respond to the needs of children and their families through an anticipatory, preventive and collaborative orientation based on the experiential learning of parents through observation of their children’s behavior, and is based on eight principles, which are guides for professional work ( 9 ). 

In the field of interventions aimed at child development with parental emphasis, there is limited evidence, so there is a need to evaluate existing interventions, as well as to develop new interventions to optimize the offer to families ( 10 ). 

With the need to explore this model and raise aspects that contribute to the proposition of new interventions, this study aimed to map the characteristics of interventions for the promotion of child development that used the TP Model, due to its potential to foster a care practice advanced in different care contexts for Brazilian children and families. No review on the subject was found in a previous search in the JBI database, the International Prospective Register of Systematic Reviews (Prospero), Cochrane Database of Systematic Reviews and the Open Science Framework (OSF) platform.

## Method

### Study type

This is a scoping review. This study was developed and structured according to the recommendations the JBI ( 11 ) and the checklist Preferred Reporting Items for Systematic reviews and Meta-Analyses extension for Scoping Reviews (PRISMA-ScR) ( 12 ). The review protocol was registered on the OSF platform ( https://doi.org/10.17605/OSF.IO/RBSG4). 

For the development of the study, the following methodological steps were taken: formulation of the research question, using the Acronym PCC-Population, Concept and Context; definition of inclusion and exclusion criteria, using the PCC and defining the types of studies and sources; elaboration of a search strategy; identification of databases; search and selection of studies; data extraction and analysis; and construction of the report ( 11 ). 

### Setting

The review was carried out in eight databases: Public MedLars (PubMed), Cumulative Index to Nursing and Allied Health Literature (CINAHL), Education Resources Information Center (ERIC), Psychological Information Database (PsycINFO), Latin American and Caribbean Literature in Health Sciences (LILACS), Database in Nursing (BDENF), *Excerpta Medica* Database (EMBASE), Scopus and Web of Science. It also includes Google Scholar, DART-Europe E-theses Portal, Brazilian Digital Library of Theses and Dissertations of the Brazilian Institute of Information in Science and Technology (IBICT), Theses CAPES, Cybertesis, EthOS and Theses Open Thesis, the Brazelton Touchpoints Center website and the Brazelton/Gomes Pedro Foundation website, as sources of grey literature. 

### Study period

It was carried out between October 2022 and January 2023.

### Selection criteria

The review question was: “what are the characteristics of interventions with parents/caregivers and children to promote child development, from pregnancy to six years of age, based on the Touchpoints Model?” The PCC Acronym ( 11 ) was defined as: P- Parents/caregivers and children; C- Interventions to promote child development that use the Touchpoints Model; C- All contexts of child care. 

Studies that responded to the research objective and the review question were included, reporting on an intervention aimed at promoting child development, from pregnancy to the child’s six years of age, and based on the Touchpoints Model. It could be an intervention already applied, or its protocol/design, and in any context of care - health, education, social assistance or home services.

Interventions were defined as activities carried out as planned, with the aim of producing a positive effect ( 4 ^,^ 13 ), whether policies, programs or individual practices. The gestation period until the child`s 6 years of age has been the focus of programs and policies in Brazil ( 14 ) and is the period covered by the TP Model ( 9 ). 

Studies that did not include the population, concept and context of interest, or that did not report the application of an intervention, such as editorials, letters to the editor and opinion articles, were excluded; in addition to incomplete studies that were not located in full after trying to contact the Brazelton Touchpoints Center and the Brazelton/Gomes-Pedro Foundation. Duplicate documents were considered only once.

There was no time, geographic or language restriction.

### Study variables

The study variables were those recommended by the JBI ( 11 ): authors; country; year of publication; kind of study; goal; population; context in which the interventions were carried out; plus those recommended by the Template for Intervention Description and Replication (TIDier) ( 15 ): theoretical framework used; professional responsible for the intervention; intervention content; materials and strategies used; delivery mode; volume (number of times, duration and period); intervention results/effects. 

### Instruments used for data collection

The standard form used for data extraction was prepared in Microsoft Word ^®^ software based on the items that must compose the interventions according to the TIDieR ( 15 ). 

TIDieR aims to improve the complete description of interventions to allow their replicability. It contemplates the following items of an intervention: why (theory); what and which (materials and strategies); who did it (professional); how (in person, via internet or telephone, in groups or individually); where (place where it occurred); when and how much (volume - number of times and what period of time); how well (whether adherence or fidelity was assessed, how and by whom; whether strategies were used to maintain and/or improve the intervention). Fidelity refers to the degree to which an intervention was delivered and received by participants as planned ( 15 ). 

The form was completed by two reviewers and a consensus was reached between the collected information and the grouping of information in a single table.

### Data collection

One of the reviewers was trained by JBI Brazil to conduct reviews and shared the guidelines learned with the other reviewer before starting the study.

Searches in the databases were carried out on October 12, 2022 through registration in the *Portal de Periódicos* (Journals Portal) of the Coordination for the Improvement of Higher Education Personnel (CAPES), via the Federated Academic Community (CAFe). Thesis databases and institutional websites were accessed on the aforementioned date via Google search engine. The definition of the databases and the search strategy was carried out with the help of a librarian. The selected descriptors were combined according to the characteristics of each database and search engine. 

The selected databases and respective search strategies are shown in Figure 1. 

**Figure 1 -:** Search strategy in databases and grey literature. São Paulo, SP, Brazil, 2023

Database and grey literature	Search strategies
PubMed ^*^	((child development[MeSH Terms]) OR “child development”[tw]) AND (touchpoints)
CINAHL †	(MH “Infant Development” OR MH “Child Development” AND TX touchpoints) AND (S1 OR S2)
ERIC ^‡^	touchpoints AND (“infant development” OR “child development”)
PsycINFO ^§^	Index Terms: {Infant Development} OR {Childhood Development} AND Any Field: touchpoints
LILACS ^||^	child development [Palavras] and touchpoints [Palavras]
BDENF ^¶^	child development [Palavras] and touchpoints [Palavras]
EMBASE ^**^	touchpoints AND (‘child development’/exp OR ‘child development’ OR ‘toddler development’ OR ‘infant development’/exp OR ‘infant development’) AND [embase]/lim NOT ([embase]/lim AND [medline]/lim)
Scopus	( TITLE-ABS-KEY ( touchpoints ) AND TITLE-ABS-KEY ( infant AND development ) OR TITLE-ABS-KEY ( child AND development ))
Web of Science	TS=(touchpoints AND (“child development” OR “infant development”))
Google Scholar	child development” AND “touchpoints model” (filtro - excluir citações)
DART-Europe E-theses	Brazelton AND Touchpoints
*Biblioteca Digital Brasileira de Teses e Dissertações do IBICT* ^††^	Brazelton AND Touchpoints
*Teses* CAPES	Brazelton AND Touchpoints
Cybertesis	Brazelton AND Touchpoints
EthOS and Theses (The British Library)	“Touchpoints AND Child Development”
Open Thesis	“touchpoints AND (”infant development” OR “child development”)”

*PubMed = Public MedLars; †CINAHL = Cumulative Index to Nursing and Allied Health Literature; ^‡^ERIC = Education Resources Information Center; ^§^PsycINFO = Psychological Information Database; ^||^LILACS = *Literatura Latino-Americana e do Caribe em Ciências da Saúde*; ^¶^BDENF = *Base de Dados em Enfermagem*; **EMBASE = *Excerpta Medica* Database; ^††^IBICT= *Instituto Brasileiro de Informação em Ciência e Tecnologia*

The search on the Brazelton Touchpoints Center and Brazelton/Gomes Pedro Foundation sites was carried out without the need for a search strategy, since all publications refer to the model. A hand search of references of the studies selected in the research was also carried out.

The results obtained from all search platforms were exported to Rayyan ^®^ (online software used for selecting studies in knowledge synthesis methods) to exclude duplicate studies and carry out the selection of searches. Two reviewers independently assessed titles and abstracts, and eligible studies were assessed in full. In case of disagreement, a third reviewer was called to settle. 

### Data processing and analysis

The data extraction form prepared in Microsoft Word ^®^ software allowed the synthesis, interpretation of data and numerical analysis of the distribution of studies, being represented by a narrative summary of the results, which were related to the research objective and question. For the description of the review, PRISMA-ScR was used. 

### Ethical aspects

There was no need to submit the study to the Research Ethics Committee.

## Results

**Figure 2 -:**
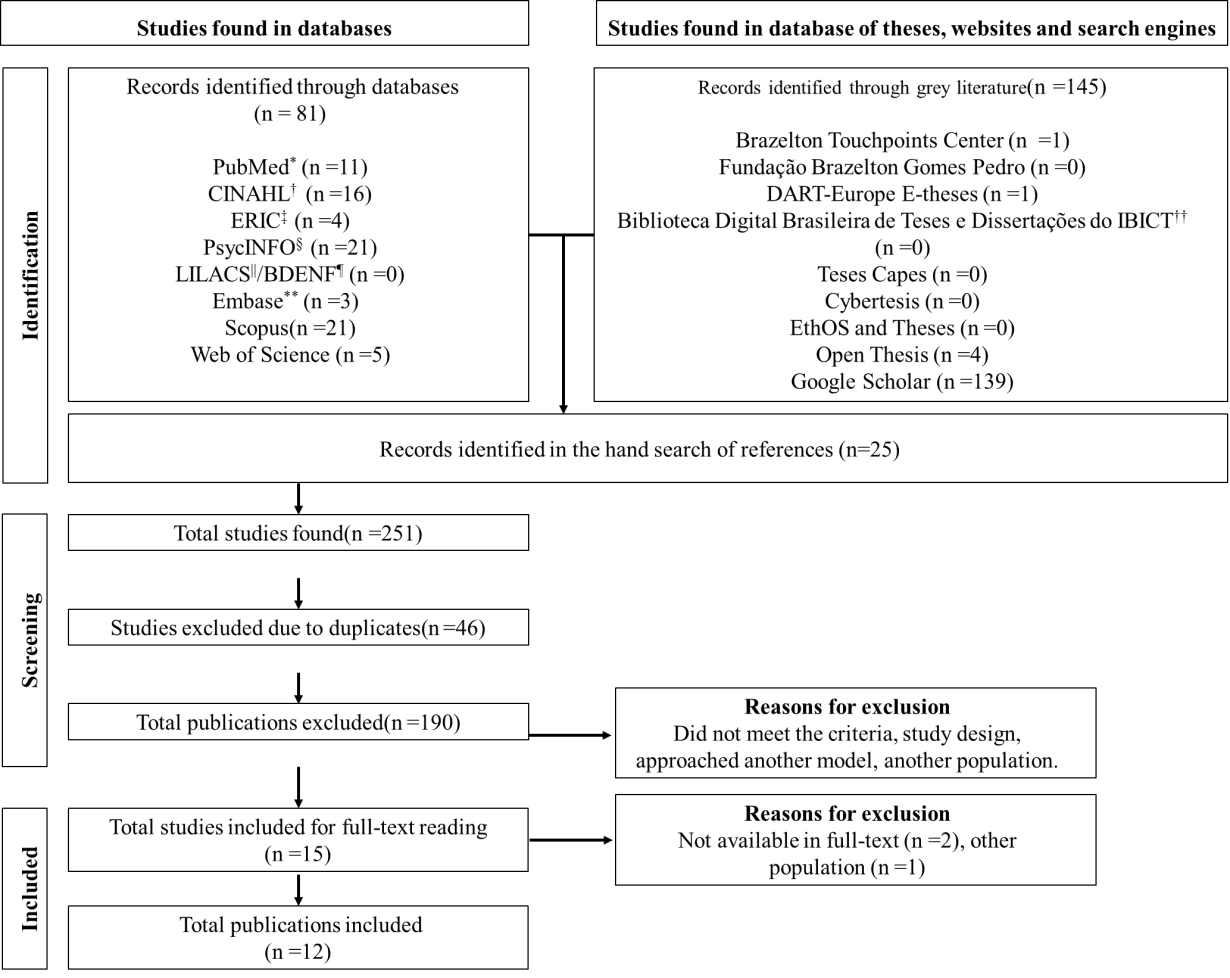
Flowchart of the article selection process for the adapted scoping review of the Preferred Reporting Items for Systematic reviews and Meta-Analyses extension for Scoping Reviews (PRISMA-ScR). São Paulo, SP, Brazil, 2023

A total of 251 studies were identified, excluding 46 duplicates via Rayyan ^®^, leaving 205 studies. A total of 190 studies were excluded by reading the title and abstract, and another three exclusion during the full-texts reading, two of them for not having been located and one for not contemplating the study population. Thus, 12 studies were included in this review: five scientific articles, one book chapter, three dissertations, and three theses ( Figure 2). 

In three of the included studies, the intervention has not yet been applied ( 16 ^-^ 18 ), therefore, there are no results/effects described. 

Most interventions have been carried out in the last twenty years in the United States ( 18 ^-^ 22 ) and in Portugal ( 16 ^-^ 17 ^,^ 23 ^-^ 26 ), in Primary Care Services ( 16 ^-^ 17 ^,^ 20 ^-^ 21 ^,^ 26 ), in school ( 19 ), in shelters ( 18 ), in hospitals and maternity ( 23 ^,^ 27 ), in the families’ homes ( 20 ^-^ 22 ) and in daycare centers ( 24 ^-^ 25 ). The identification of the studies is presented in Figure 3. 

*PubMed = Public MedLars; †CINAHL = Cumulative Index to Nursing and Allied Health Literature; ^‡^ERIC = Education Resources Information Center; ^§^PsycINFO = Psychological Information Database; ^||^LILACS = *Literatura Latino-Americana e do Caribe em Ciências da Saúde*; ^¶^BDENF = *Base de Dados em Enfermagem*; **EMBASE = *Excerpta Medica* Database; ^††^IBICT = *Instituto Brasileiro de Informação em Ciência e Tecnologia*

**Figure 3 -:** Identification of studies according to author, country, year of publication, type of study and objective. São Paulo, SP, Brazil, 2023

Author/Country/Year	Study type	Objectives
Percy MS, et al. ( 19 ) United States. 2001	Pilot study	To test an intervention that aims to increase parental self-confidence among pregnant women and low-income teenage mothers.
Farber MLZ ( 20 ) United States. 2009	Pilot, quasi-experimental study	To strength anticipatory guidelines for promoting child development in Latino and African-American families in a Primary Care Center; strengthen the adequacy of family needs and resources, increase families’ knowledge about educational practices and promote resilience; promote immunization and child development.
Guthrie KF, et al. ( 21 ) United States. 2009	Randomized controlled trial	To verify whether short-term interventions with high-risk pregnant women can improve parental attitudes and home environments.
Brandt K, et al. ( 22 ) United States. 2010	Prospective and retrospective randomized study	To verify whether a TP* model-based approach to public health nurse home visits is associated with better six-month outcomes for high-risk dyads than a traditional nurse home visit model or no home visit.
Vilaça S, et al. ( 23 ) Portugal. 2012	Randomized controlled trial	To analyze the effect of the health education program for mothers (PEPDI - Parental Empowerment Program for Child Development), on child development, as well as on maternal characteristics.
Castelão ASD ( 25 ) Portugal. 2013	Action-research	Study the implementation of a Touchpoints program to promote a relationship of trust and partnership between the teacher and the parents.
Pinto RMP ( 24 ) Portugal. 2013	Action-research	To study the impact of Touchpoints training on the educator’s representations and in the educator-parent relationship, and to verify how alterations in the educator’s representations and in the relationship established with the parents affect the child’s development and the quality of the educational context.
Soares HM ( 26 ) Portugal. 2016	Quasi-experimental longitudinal study	To evaluate the impact of clinical nursing intervention using the TP* model on mother-child interactive behavior and child development between the first and second year of life.
Martins PAC ( 17 ) Portugal. 2017	Pilot study †	To promote child development and parenting in the first year of life.
DiCero KE ( 18 ) United States. 2018	Pilot study †	To reduce or prevent the involvement of families in the Massachusetts Department of Children and Families, increase the mother’s positive attachment interactions with her baby; document a typical developmental trajectory for babies in the program; achieve positive connections of adolescent mothers with the therapist.
Fareleira F, et al. ( 16 ) Portugal. 2021	Randomized clinical trial protocol †	To evaluate the effect of a parental intervention based on the Touchpoints model on the sense of parental competence of parents of children younger than 18 months, in comparison with usual care and its relationship with other dimensions: mental health and parental well-being (stress, depression, anxiety, psychological experience of pregnancy, attachment, quality of life), child development (physical/sensory-motor, psychosocial) and family well-being (family and couple functioning).
Shimpuku Y, et al. ( 27 ) Japan. 2022	Longitudinal, quasi-experimental study	To determine whether mothers who receive HUG Your Baby prenatal education do better than a control group with regard to postpartum depression and related factors.

*TP = Touchpoints; †Research in which the intervention has not yet been applied

The nurse was the professional who performed the intervention in six studies ( 19 ^-^ 23 ^,^ 26 ), in one of them together with researchers ( 19 ) and, in another, with physicians ( 21 ); will also be the professional responsible for two of the interventions not yet carried out ( 16 ^-^ 17 ). The other responsible professionals were physicians ( 16 ^-^ 17 ^,^ 21 ), researchers ( 16 ^,^ 19 ^,^ 27 ), educators ( 20 ^,^ 24 ^-^ 25 ) and social worker ( 20 ). 

The period of the intervention, duration and frequency differed between studies. Two of the studies carried out the intervention in person, in groups ( 19 ^,^ 27 ), six performed face-to-face and individually ( 21 ^-^ 26 ), and one study carried out in a hybrid format, with face-to-face meetings and telephone contact for follow-up ( 20 ). Of the interventions not applied, one will be in a group ( 18 ) and two will occur individually ( 17 ^-^ 18 ). 

The main contents worked on in the interventions were the experience of the parents during pregnancy and labor ( 16 ^-^ 17 ^,^ 19 ^,^ 21 ^-^ 22 ^,^ 24 ^,^ 26 ); the change in the relationship with the family after the baby’s birth; the difficulty of fatherhood; the child’s temperament ( 19 ); strengthening the parent-health-professional relationship with a focus on child development, parent-child interaction, appropriate behaviors for the child’s age; increased knowledge of families and use of community resources ( 20 ^-^ 21 ^,^ 23 ^,^ 27 ); strategies for developing parental sensitivity and understanding of child development ( 17 ^,^ 20 ^-^ 21 ); anticipatory care adjusted to key ages based on the Touchpoints Model ( 16 ^-^ 21 ^,^ 23 ); child’s developmental skills ( 21 ^,^ 26 ); situations predictable to occur in relation to development, challenges and parenting concerns ( 18 ^,^ 23 ). 

Changes in the intervention during the research were described in only one study, with the inclusion of night classes for spouses and boyfriends at the request of mothers ( 19 ). Two studies offered incentives for participation, such as toys and books, calendars, certificates and gifts from grocery stores ( 20 ) and $50 shopping vouchers ( 21 ). Adherence to the intervention was described in only one study, which did not describe the strategies used to maintain or improve adherence ( 19 ). Figure 4 details the characteristics of the interventions. 

**Figure 4:** Characterization of interventions. São Paulo, SP, Brazil, 2023

Population	Theoretical Framework	Materials used	Strategies used	Responsible Professional	Way of delivery	Context	Volume	Results//Effects
20 pregnant or teenage moms and dads ( 19 ).	Touchpoints Model	Not described	Mothers participated in classes and discussions about the experience of pregnancy and childbirth, baby behavior, child development and anticipatory care.	Pediatric nurse, professor with experience with children and adolescents and trained in the TP* model, researchers, doctoral student	Face-to-face and in groups of 20 mothers	School in rural area	1 time a week, lasting 1 hour, totaling 15 weeks.	There was a significant increase in parental self-confidence after the intervention. The biggest changes were observed in items addressing the decrease in frustration in caring for babies and the degree of comfort with motherhood.
35 family members. mothers with 17 years or older at the birth of the baby. All immigrants from Central and South America and Puerto Rico; and children between 16 and 18 months ( 20 ).	Touchpoints Model and Transactional Model of Child Development	For parents, we used 5 handouts and videos about CD †, interactions, meaning of behaviors, and anticipatory care; and the Ages and stages Questionnaire to help them assess, understand and anticipate their child’s development, as well as books and toys. For professionals, 6 instructional videos were used on behavior, signs, temperament, feeding interaction and communication between staff and parents, and study guides for reflection.	Families received a Primary care center, home visits from birth to 18 months of age, and telephone contacts. The focus of the meetings was CD † and parent-child interaction. The professionals received a week’s training in the TP* model.	Social work clinical research director, health center nurse director, education director, parent trainers, supervisor trainers, project manager, data manager, and bilingual (English and Spanish).	For families: hybrid format, face-to-face and individual, and follow-up via telephone	Primary care center; home visits	The visits began at the birth of the child and continued until she turned 18 months old. The first visit lasted two hours. No further information specified..	There was a strengthening of the families’ global resources, such as time and care for the children, use of age-appropriate toys, understanding of the child’s needs and development. The use of the Ages and Stages Questionnaire was seen by parents as a way to promote understanding of the child’s development and socio-emotional needs. The children were immunized following the schedule and showed adequate development, with the exception of 3, who were referred to the stimulation service.
66 high-risk pregnant women and their babies. 33 from IG ^‡^ and 33 from GC ^§(^ 21 ).	Touchpoints Model	Recorded tapes of parent-baby interactions	The IG ^‡^ received visits by professionals trained in the TP* model. The GC ^§^ received a visit when the baby was 3 months old, without using the TP* model approach.	Residents, physicians, and nurses	Face-to-face and individual	Family Clinics and Home Visits	IG ^‡^: one hospital visit at birth. Home visits: twice a month for one hour until the baby is 3 months old (6 visits). GC ^§^: a visit when the baby is 3 months old	IG‡ scored higher on the Adult–Adolescent Parenting Inventory (AAPI) and significant differences in 2 of the 6 IT-HOME ^||^ domains: responsiveness (p ^¶^ = 0.05) and learning materials (p ^¶^ = 0.05). Responsiveness included praising the child, showing affection, and reacting positively to the child’s vocal expressions. Learning materials included toys and other developmental items.
70 high-risk pregnant women. 35 of them from the “baseline” group, 15 from the comparison group and 20 from the IG ^‡(^ 22 ).	Touchpoints Model	Not described	IG ^‡^ professionals received training in the TP* model and monthly reflective practices. The “baseline” group did not receive visits from the nurses; the comparison group received visits from nurses not trained in the TP* model and the IG ^‡^ received visits from nurses trained in the TP* model.	Public health nurses trained in the TP model*	Face-to-face and individual	Home visits	The comparison group received 5 visits and the IG ^‡^ received 7 visits lasting 30 to 60 minutes. Intervention period was not specified.	The IG ^‡^ presented better health and developmental outcomes for the baby, with fewer ER visits, more childcare visits, longer duration of breastfeeding and greater satisfaction with services. The VDs** of nurses trained in the TP* model were the strongest predictor of better outcomes.
411 of mother/baby dyads, with babies born at term, eutocic delivery, Apgar score greater than 7. 205 in GC ^§^ and 206 in IG ^‡(^ 23 ).	Nola Pender’s Health Promotion Model and Touchpoints Model.	A protocol was used as a guide for each session, including topics for discussion, objectives and what to evaluate and expected learning outcomes.	The intervention was carried out through of parental training in the perspective of health promotion. Contents of each phase of development, feeding, anticipatory care and parental concerns were addressed. In the GC ^§^, standard care was provided to monitor development.	Nurses	Face-to-face and individual	Hospital obstetric ward	4 sessions: 1 ^st^ week of life, 2 ^nd^, 4 ^th^ and 6 ^th^ month of life. Each session lasted 40 minutes	The development indices of the children in the IG ^‡^ were significantly higher (p ^¶^ < 0.001) than the CG ^§^ indices. Maternal anxiety rates were significantly lower (p ^¶^ < 0.001) in IG ^‡^. Also showed a significant effect for this decrease the increase in maternal knowledge (p ^¶^ < 0.001), belonging to low socioeconomic level compared to high, medium-high and medium (p ^¶^ = 0.006).
11 parents of 12 children aged between 22 and 36 months, who attended the day care center ( 25 ).	Bronfenbrenner’s Touchpoints Model and Bioecological Theory	Reflective sheets, maps of weekly routines and videos of parent-child interaction.	The intervention consisted of training in the TP* model with the educators, reflective practices and the elaboration of a reflective diary for 6 weeks and evaluation of the mother-child interaction through a 3-5 minute recording of playing situations, before and after the training of educators.	Educator responsible for the study, Touchpoints team, early childhood educator at the same institution	Face-to-face, individual (interviews) and group (training) (professionals). The families was not described.	Nursery	Completion of the reflective practice form: weekly. Elaboration of the professionals’ reflective diary: 6 weeks. The evaluation of the educational context took place over 3 hours. The interview with the classroom educator: 30 minutes. The families’ routine map: one week (before and after PT* training).	Child development, parent-child relationships, family routines, parent-teacher and parent-teacher satisfaction, as well as the educator’s own practice, changed after training in TP* and reflective practices. The child showed significantly higher values in all dimensions of the Child Development Scale Growing Skills II, there was greater interaction between parents and children and a greater number of tasks performed by parents.
11 families and 11 children aged between 13-33 months, who attended daycare ( 24 ).	Touchpoints Model and Bronfenbrenner’s Bioecological Theory	Reflective practice sheets filled out by educators weekly (before and after training in the TP model*) were used, and maps of weekly routines prepared by families, which included the routine, who performed the routine, the time of the routine, how the routine went , and were completed before and after the application of the TP* model by the educators. Parent-child interaction videos were also used.	The intervention consisted of training in the TP* model with the educators, reflective practices and the elaboration of a reflective diary for 6 weeks and evaluation of the mother-child interaction through a 3-5 minute recording of playing situations, before and after the training of educators.	Educator responsible for the study, Touchpoints team, and early childhood educator at the same institution	For professionals: face-to-face, individual (interviews) and group (training) Paras the families was not described	Nursery	Reflective practice form: carried out weekly, before and after training. The professionals’ reflective diary took place for 6 weeks. The evaluation of the educational context took place over 3 hours. The observation, the interview with the classroom educator lasted 30 minutes. The families’ routine map: one week (before and after PT* training).	The child showed significantly higher values in all dimensions of the Child Development Scale Growing Skills II, with less difference in the vision and autonomy dimensions (0.50). Significant differences were also found in parent-child interaction, assessed by the Care-Index scale. The number of chores performed by mothers decreased slightly, and the number of tasks dads performed doubled.
2 nurses, 86 healthy children aged up to 11 months and their mothers, 43 children and mothers from IG ^‡^, and 43 from CG ^§^ (standard health follow-up) and 40 without intervention and follow-up ( 26 ).	Touchpoints Model	The family needs inventory was used for them to indicate topics they would like the team to evaluate and discuss, such as: growth and development, playing, how to deal with concerns about CD †, aspects of social, family and financial support.	The IG ^‡^ families received HV ^††^ with the TP* model approach, and the CG ^§^ received visits at the same age as the children, without using the TP* approach.	Nurses of Primary Care services	Face-to-face and individual	Primary care center	4 follow-up visits: 11 months of the child, 12 months, 18 months 24 months. Time of visits not specified.	The intervention had a positive effect on the dimensions: development, maternal representations about the child and motherhood, maternal sensitivity, child cooperative behavior and perception of trust in relation to nurses. Parents considered that the TP* model contributed to the acquisition of knowledge and skills, validation of parenting practices, parental trust, interpersonal relationships and satisfaction.
Multiprofessional team, nurses, children in the first year of life and their families ( 17 ).	Betty Neuman’s Systems Model, Family-Centered Care, and Touchpoints Model.	Use of a manual on anticipatory care, leaflets with activities that promote child development at each age, and the Newborn Behavioral Observations (NBO) to promote bonds and parental competence.	The intervention will be carried out in the development evaluation consultations. Anticipatory care will be addressed based on the TP* model for each age.	Nurse	Face-to-face and individual	Primary care center	6 consultations: 1 month, 2 months, 4 months, 6 months, 9 months, 12 months. The time of consultations was not described.	Not applicable. Intervention not yet performed
Teenage mothers with and without mental illness living in shelters and their babies ( 18 ).	Dyadic therapy; Touchpoints Model; Floortime; Mutual Regulation Model and Shared Dyadic States of Consciousness.	Videos from YouTube and DVD ^††^ will be used about the baby’s first years, baby’s behaviors and temperaments, teaching boundaries with love; songs, a book on how relationships support development and another on baby behavior, children’s toys, handouts for families	The intervention consists of didactic therapy with the mothers and babies, which will take place in groups of 4 dyads, and in individual meetings. Classes will also be taught with a group of ten dyads, using the TP Model approach* and focusing on anticipatory care and the developmental needs of babies.	Child mental health physician, trained in development	Face-to-face, individual and in groups of 4 dyads for day therapy and 10 dyads for classes	Shelter	Group meeting lasting 90 minutes *per* week for a period of six weeks. Mother-baby meeting, weekly, lasting 90 minutes. Classes: 1 hour *per* week for 15 weeks. 60 minutes for dyadic therapy and 30 minutes for scaffolded playtime	Intervention not yet performed
Three family doctors and one nurse per unit. 216 parents (from pregnancy to 18 months of the baby) ( 16 ).	Touchpoints Model	For families, 28 leaflets on anticipatory care will be used for each Touchpoint, addressing needs and fears. For the training of professionals, slides, videos and leaflets will be used.	IG ^‡^ parents will receive consultations with the TP model approach*. The CG§ will receive routine care from Primary Care, without the TP* approach. IG ^‡^ professionals will receive training in the TP* model lasting 5 hours	For families: physicians and nurses trained by the researcher For IG ^‡^ professionals: Researcher	For families: face-to-face and individual. For the professionals: face-to-face and in groups	Maternal and Child Primary Health Care Center	Each family will receive approximately six prenatal consultations and nine childcare consultations. Consultation time not specified.	Not applicable (Protocol)
221 Pregnant women, after 30 weeks of gestation. 121 of the IG ^‡^ and 121 of the GC ^§(^ 27 ).	Neonatal Behavioral Rating Scale (NBAS) and Touchpoints Model.	A PowerPoint presentation, a 20-minute HUG Your Baby video, a guide to successful breastfeeding, a booklet on child development, dolls for the practice of swaddling (swaddling the baby), handout with script for practice and fabric to swaddle it at home.	IG ^‡^ participated in classes that addressed the child’s behavior, breastfeeding, CD ^||^, newborn sleep-wake cycles, how to respond to stressful situations in the baby, and safe swaddling practices *.*	Researchers	Face-to-face and in a group of 15 mothers	Childbirth Center prenatal clinic, Maternity and University classroom	The intervention lasted approximately two hours. Periodicity was not described.	There were significant differences between the two groups with regard to: knowledge of the infant’s behavior (baseline, one month and three months) (p ^¶^ < 0.01), Karitane Parental Confidence Scale (KPCS) scores at one month (p ^¶^ <0.01). The intervention demonstrated a positive impact on preventing postpartum depression, increasing parental confidence, reducing maternal stress, and increasing knowledge about the baby’s attachment

*TP = Touchpoints; †CD = Child development; ^‡^IG = Intervention group; ^§^CG = Control group; ^||^IT- HOME = Infant - Toddler HOME Inventory; ^¶^p = Significance Level; **HV = Home visit; ^††^DVD = Digital Versatile Disc

## Discussion

Intervention programs are considered essential for understanding the behavior and needs of children by parents, promoting parenting competence, learning about child development and greater stimulation of children ( 4 ^,^ 6 ^,^ 28 ^-^ 30 ). Thus, this review mapped the characteristics and effects of interventions that used the Touchpoints Model with emphasis on the essential components described in TIDieR ( 15 ). By mapping the complete description of the interventions, it becomes possible to identify characteristics that provide the achievement of favorable results, to implement them, replicate them, or develop new studies based on the findings. 

There was a concentration of production in the United States, possibly because it is the country in which the model was created, and because of the existence of the Brazelton Touchpoints Center (BTC), which promotes training for professionals ( 9 ), and in Portugal, for hosting a BTC partner foundation, the Brazelton/Gomes Pedro Foundation, which works to disseminate a clinical intervention paradigm based on the model ( 31 ). 

The first reflection about the studies is about the variability of interventions with regard to content, strategies and volume. Figure 4 shows that there is repetition of only one intervention ( 25 ^-^ 26 ). In the others, although some elements are recurrent, the complete design of each one is always new. This occurs because they are designed according to research objectives, which focus on specific aspects of expected results. It is observed, therefore, that there was no particular intervention tested in multiple aspects, even though all were based on the Touchpoints model. The discussion on the constituent elements of the interventions, below, sheds light on aspects that can contribute to the understanding of such interventions, based on common points and variability. 

The predominance of nurses as responsible for applying most interventions is in line with this professional’s profile. Studies have highlighted the essential role of nurses in empowering parents through strategies that allow exploring parenting, incorporating knowledge about CD ( 28 ^-^ 29 ), support the construction of the parental role, and understand the reality of each assisted family, for physical health care and to promote a safe environment ( 32 ). Thus, although the Touchpoints model appears to be appropriate for the different professionals who work with children, it is possible that studies have prioritized nurses in carrying out interventions due to this broad profile. 

As for the contexts of application, the predominance of consultations in Primary Health Care (PHC) and home visits (HV) can synergistically contribute to actions based on the model. PHC is a favorable environment with infrastructure to implement interventions to promote child development in the monitoring of children’s health in the early years ( 7 ), and scientific evidence shows that home visits offer favorable conditions for working on early childhood issues and needs, with better development of children in cognitive, behavioral and socio-emotional dimensions ( 29 ^,^ 33 ). 

As for the various resources used in the interventions, such as leaflets, videos, guides/scripts, handouts, books and toys, they are similar to those usually used in research on educational interventions ( 4 ^,^ 7 ^,^ 30 ). However, the absence of information about the intervention material is remarkable, given that it was available in only two studies, one that shared the folder used ( 27 ), and another that provided the Uniform Resource Locator (URL) of the videos and songs ( 18 ). This makes it difficult to objectively appreciate such materials and even relate the results to their quality. Still, it does not allow interventions to be replicated in other contexts for new evaluations. 

The main contents explored in the interventions were the parents’ experience during pregnancy and labor; parent-child interaction; child’s behaviors as their language; understanding of child development; anticipatory care for every age; parenting challenges and concerns. These contents are in line with the main topics to be addressed from the prenatal Touchpoint ( 9 ), showing fidelity to this aspect of the model. However, as previously mentioned, it was not possible to access the contents of the interventions in a complete way, which makes it impossible to measure whether the interventions follow all the principles and assumptions, guaranteeing this fidelity. 

Although the volume of the intervention differed between the studies, ranging from six weeks to 18 months, and, in most cases, there is no description of the duration of each meeting, nor the fidelity of the intervention, that is, whether it was delivered as planned or recommended by the model ( 15 ), all had positive results. This shows that the effects on parental behaviors, knowledge and attitudes can be quickly visible when using an approach that values disorganization and vulnerability as an opportunity to support and validate parenting practices, rather than treating them as weaknesses or harmed behaviors. 

Review studies have shown good effectiveness of interventions focused on parenting to promote development ( 4 ^,^ 34 ), although another review points out the infeasibility of performing meta-analyses or meta-syntheses of studies of this nature, or understanding why the interventions work, due to the variability of the interventions tested ( 10 ). Adherence was mentioned in only one study ( 19 ), which did not detail the strategies used to maintain or improve it. 

Fragility is observed in the description of these central constructs of the interventions, which was also identified in a systematic review and meta-analysis that evaluated the effectiveness of parental interventions ( 4 ). This lack of transparency makes it difficult to carry out interventions on a large scale, as it does not allow for the understanding of more or less successful interventions, and the dissemination of what worked or not, for whom and how ( 13 ). 

Regarding the mode of delivery, a greater offer of interventions individually, to the detriment of group interventions, shows a possible preference of researchers for the individual approach. This contradicts current research that points to the widespread use of group strategies, as they promote learning among peers through the exchange of knowledge and sharing of experiences, frustrations and anxieties ( 6 ^-^ 7 ^,^ 30 ). 

The use of current technologies in interventions was not identified, such as the internet (message sending applications, videoconferences, social media), and only one of them was carried out in a hybrid format, with face-to-face meetings and telephone follow-up ( 20 ); this may be related to the fact that most studies were conducted more than 10 years ago. Currently, technologies are part of everyday life for families and enhance communication between health professionals and parents, facilitating and favoring different ways of delivering interventions, although there is the challenge of disparities in access to these technologies ( 7 ). 

The interventions did not explore the TP Model in depth, did not describe how it was used in its design, and which principles were applied. However, in the description of contents and effects, three principles were identified in most studies: “use the child’s behavior as their language, focus on the parent/child relationship and look for opportunities to support parental dominance” ( 9 ). 

All interventions carried out qualification/training in the TP model for the professionals who applied the interventions with the target audience, but there is no information on how or if the consistency of professionals in working with families was verified.

In the present review, the strategies used in the interventions were directed towards changing the knowledge, behavior and attitude of the parents in order to understand the development and behavior of their children. Research identified the paradigm shift proposed by the TP model, focused on anticipatory guidance and collaborative practice based on the experiential learning of parents ( 9 ). 

The main outcomes of the interventions were better global CD results, greater understanding of CD and the child’s behavior as its language, improved sense of parental competence and greater maternal sensitivity, greater interaction, responsiveness and time with the child, greater use of toys and learning materials, resembling the results of other parenting interventions ( 4 ^,^ 6 ^,^ 28 ^-^ 30 ^,^ 34 ). 

It was possible to examine the nature and extent of the interventions that used the TP model, and, although the positive effect is evidenced in all studies, the weakness in the description of the volume does not allow relating them to the duration of the intervention, and the heterogeneity of the interventions limits the comparison of these results.

As the main contributions of the study, one can consider the positive outcomes present in all investigations, in addition to highlighting the importance that reports of intervention studies contemplate in a more comprehensive way the description of the intervention itself.

Although the positive outcomes may justify the recommendation to test interventions based on the TP model in the Unified Health System (SUS), it is only possible to consider that they should include the common characteristics described in the studies, such as the anticipatory care approach, guidance on child development and parenting, carried out by nurses and in PHC. However, the lack of details on the interventions does not allow us to state that such positive outcomes, even in short-term interventions, are related to the non-prescriptive approach and other characteristics of the professional-family relationship that differentiate the TP model from traditional interventions.

Even so, such results justify further studies for the use of the model in public policies in Early Childhood, to support practices that promote CD, with emphasis on the socio-emotional, as it is innovative, when considering Touchpoints instead of just developmental milestones, and when acting from the perspective of family-child and professional-family interactions ( 9 ). 

Although no time or language cut-off was carried out, the limitations of the study refer to the small number of publications located, mainly in the last five years, with a predominance of studies in the grey literature to the detriment of the nine databases, and the exclusion of two searches due to not being located. Still, the incomplete description of the key components of the interventions makes it difficult to understand the results and compare them.

## Conclusion

Interventions focused on the period from pregnancy to three years of age, and most were carried out by nurses in Primary Care and in the HV, prioritizing the dissemination of Touchpoints content according to age, and aspects of parenting. The studies showed potential for favorable results for child development and parenting. The variability of interventions made it difficult to map the most effective characteristics.

The mapping of interventions that used the TP model responds to the need in the field of parental interventions and explores a model that can be used and better characterized in new interventions to compose public policies in Early Childhood, given the positive results observed so far.
